# SULF1 in Cancer Associated Fibroblasts Promotes Invasion in Head and Neck Cancer Cell Lines

**DOI:** 10.1002/cam4.71540

**Published:** 2026-01-18

**Authors:** Pritha Mukherjee, Julius Benicky, Aswini Panigrahi, Laurie Ailles, Radoslav Goldman

**Affiliations:** ^1^ Department of Oncology Lombardi Comprehensive Cancer Center, Georgetown University Washington DC USA; ^2^ Clinical and Translational Glycoscience Research Center Georgetown University Washington DC USA; ^3^ Department of Medical Biophysics University of Toronto Toronto Ontario Canada; ^4^ Department of Biochemistry and Molecular & Cellular Biology Georgetown University Washington DC USA

**Keywords:** 3D spheroid coculture, cancer cell invasion, cancer‐associated fibroblasts (CAFs), CRISPR/Cas9 gene editing, SULF1

## Abstract

**Background:**

Cancer‐associated fibroblasts (CAFs) significantly influence tumor behavior in head and neck squamous cell carcinoma (HNSCC) and other malignancies. We identified the extracellular sulfatase SULF1 as a key stromal factor highly expressed in CAFs and associated with poor prognosis.

**Methods and Results:**

Using CRISPR/Cas9‐edited SULF1‐knockout primary HNSCC CAFs, we demonstrate that loss of SULF1 reduces fibroblast proliferation and markedly impairs cancer cell migration and invasion in vitro. Two‐photon microscopy in 3D spheroid cocultures revealed that SULF1‐deficient CAFs fail to support invasiveness of Cal33 cells, resulting in spheroids with fewer invasive projections and altered morphology. Proteomic analysis confirmed the absence of SULF1 in the knockout cell cultures and revealed that SULF2, expressed in tumor cells, does not compensate for its loss.

**Conclusion:**

These findings highlight the importance of CAF‐derived SULF1 in regulating tumor invasion and suggest that SULF1 is a promising therapeutic target in HNSCC.

Head and neck squamous cell carcinoma (HNSCC), which includes cancers of the oral cavity, larynx, and pharynx, ranks as the seventh most prevalent cancer globally [1]. In the United States alone, there are approximately 50,000 new HNSCC diagnoses and 12,000 deaths annually [2]. The aggressive nature of HNSCC is significantly influenced by its extensive stromal infiltrate, predominantly composed of cancer‐associated fibroblasts (CAFs) [3]. Myofibroblastic CAF‐derived signals, including various proteins, synergistically promote tumor growth, invasion, metastasis, angiogenesis, and immune evasion in multiple cancers [4, 5, 6, 7]. This multifaceted role establishes CAFs as critical drivers of cancer progression and, consequently, attractive therapeutic targets [8, 9].

Heparan 6‐O‐endosulfatases are crucial enzymes that modify postsynthetically heparan sulfation, thereby regulating the gradients of vital signaling molecules such as growth factors (e.g., FGFs, VEGFs, HGF), morphogens (e.g., WNTs, BMPs, Hedgehogs), cytokines (e.g., ILs, TGF‐betas), and chemokines (e.g., CCLs, CXCLs). These signals are essential for normal tissue development and homeostasis [10, 11]. Disruptions to these finely balanced processes can significantly impact cancer progression by altering cellular communications within the tumor microenvironment. While our previous research confirmed that the SULF2 isoform of heparan 6‐O‐endosulfatases promotes tumor cell growth and invasion in HNSCC [12, 13], we detected only minimal amounts of the SULF1 isoform within the cancer cells themselves even though SULF1 is one of the most consistently upregulated proteins in the HNSCC tumor tissues [12, 14]. Instead, we identified a predominant expression of SULF1 in CAFs within HNSCC tumors [12, 15], a finding consistent with other studies [16, 17]. In addition, we have shown that SULF1 expression is high in multiple cancers and is associated with poor survival outcomes [12]. We, therefore, generated SULF1 knockout CAFs (S1KOCAFs) and compared their ability to stimulate cancer cell migration and invasion to that of wild‐type counterparts (HNCAF37).

SULF1‐deficient primary human HNCAF37 cells were generated by Crispr/Cas9 gene editing [18] (Table S1) The population doubling time was 2.1 and 7.4 days for HNCAF37 and CAFS1KO, respectively. This 3.6‐fold change in decreased doubling time upon SULF1 knockout indicates that SULF1 is crucial for proliferation of CAFs (Table S2). To investigate whether SULF1^+^ CAFs influence tumor cell migration, we performed wound healing and migration assays using SCC35 cells (human squamous cell carcinoma cell line) exposed to different conditions (Figure 1). SCC35 cells treated with HNCAF37‐derived conditioned media (CAF‐CM) showed enhanced wound closure compared to the control (10% serum) in 24 h (Figure 1A). However, SCC35 cells exposed to S1KOCAF‐CM migrate significantly less compared to the CAF‐CM (Figure 1B). To further assess the influence of the SULF1^+^ CAF on migration of the SCC35 cells, we utilized Boyden chamber assays. SCC35 cells were seeded in migration chambers with bottom compartments containing either 10% serum (Condition1), HNCAF37 cells, or S1KOCAF cells (Figure 1C, upper panel). SCC35 cells displayed robust migration towards the HNCAF37‐cell chambers (Condition 2), whereas migration was significantly reduced in the presence of HNCAF37‐S1KO cells (Condition 3). We also observed that SULF2 knockout SCC35 cells (SCC35‐S2KO) migrate better towards the HNCAF37 cells compared to control, but significantly less in the presence of S1KOCAF (Figure 1C, Lower panel). This reduction is greater than that of S1KOCAF alone as seen by the area covered by the migrated cells (Figure 1D). This observation confirms that SULF1^+^ CAFs actively support local invasion of HNSCC cells, and that SULF2 in cancer cells further augments the migration process.

**FIGURE 1:**
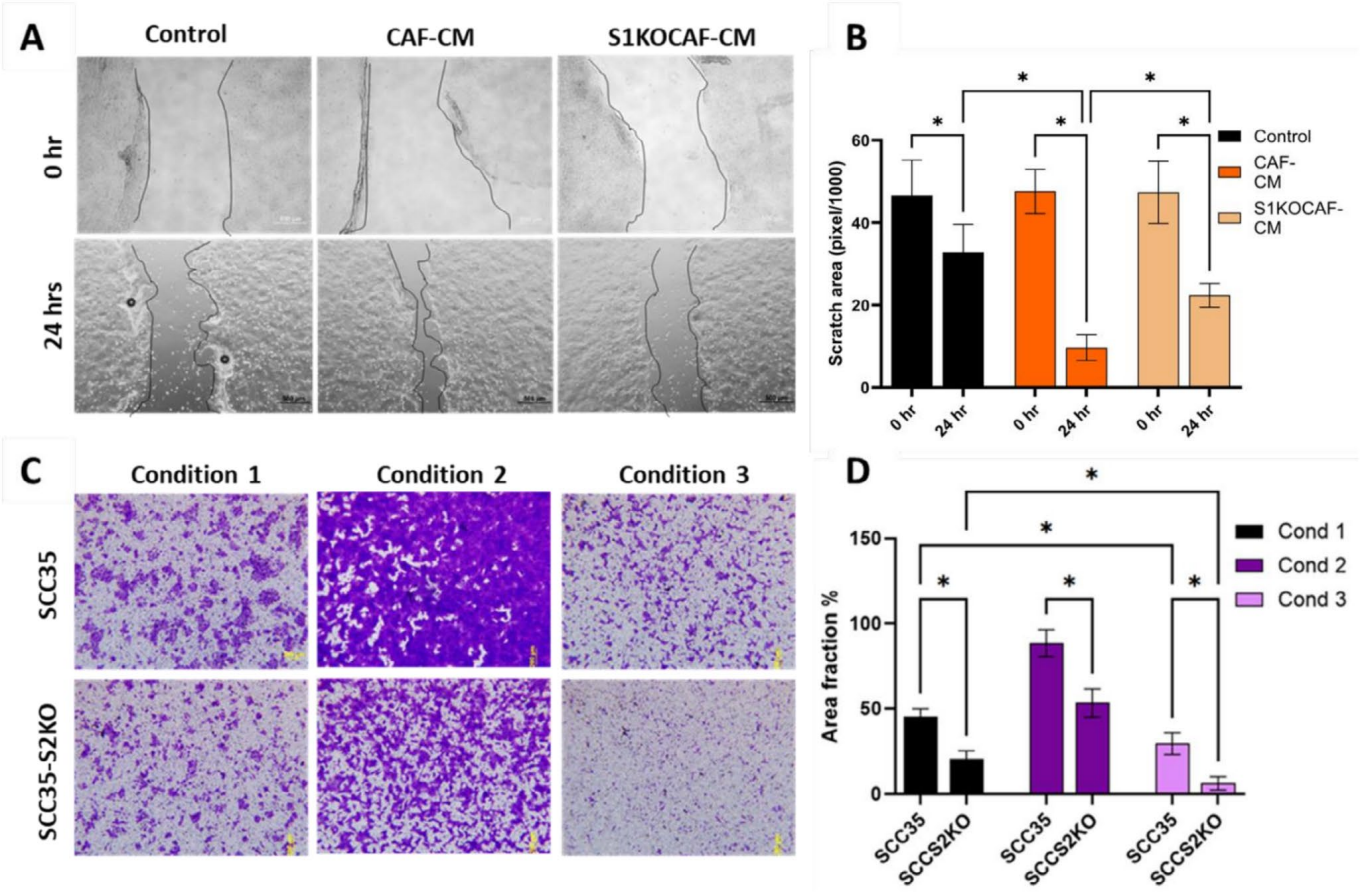
(A) Wound healing assay of SCC35 cells showing cell migration under different conditions of 10% serum (Control), cell free conditioned media from HNCAF37 (CAF‐CM) or from S1KOCAF‐CM. (B) Wound closure depicted by the scratch area in pixels (*n* = 3). (C) Migration of SCC35 and SCC35‐S2KO cells in 24 h towards bottom chambers with 10% serum (Condition 1), HNCAF37 cells (Condition 2), and HNCAF‐S1KO cells (Condition 3). Cell migration in the Boyden chamber is indicated as percent area covered. (D) Graphs represent the median ± SD (*n* = 3) for every condition measured. Statistical significance was assessed using one‐way ANOVA. * indicates *p* ≤ 0.05.

In order to understand the effects of Sulf1^+^ CAFs on tumor cell invasion, we performed spheroid coculture assays using GFP‐tagged Cal33 and SCC35 cells embedded in Matrigel. Spheroids of each cancer cell line were grown with either HNCAF37 or the S1KOCAF cells at a 1:1 cell ratio, and their morphological features were monitored over 5 days. Fluorescence imaging demonstrated that spheroids cocultured with HNCAF37 expand and form irregular invasive protrusions significantly more than the S1KOCAF cocultures (Figure 2A). Quantitative measurements indicated lower area, inverse circularity, and fewer boundary protrusions in the S1KOCAF cocultures (Figure 2B), confirming that the Sulf1^+^ CAFs enhance expansion and invasion of the cancer cells into Matrigel. Structural evaluation of the spheroids using two‐photon confocal laser scanning microscopy further confirmed altered spatial distribution of Cal33 cells in the presence of the S1KOCAFs (Figure 2C). Spheroids cocultures with S1KOCAF have smoother boundaries without invasive protrusions, indicating abrogated invasion and lower green intensity in the spheroid core, indicating lower cell density. In addition, SULF2KO Cal33 cells resulted in spheroid cocultures with lesser area but with protrusions into Matrigel (Figure 2C). This supports our hypothesis that SULF1+ CAF alter the local microenvironment by creating invasive tracks through which the cancer cells migrate, while SULF2, originating from the cancer cells, promotes cancer cell growth more than it affects the local invasion. Relative quantification of Sulf‐1 and Sulf‐2 proteins was performed using LC/MS analysis. It confirmed substantial level of Sulf‐1 in WT cells, but the S1KOCAF cocultures have no detectable Sulf‐1 (Figure 2D). Sulf‐2 protein was present in both with an increase in KO environment (where we anticipate relative increase of cancer cells in proportion to CAF cells). This indicates that the Sulf‐2 in the cocultures did not compensate for the lack of Sulf‐1 (Figure 2D), which further supports the importance of Sulf‐1 provided by CAF in the tumor microenvironment.

**FIGURE 2:**
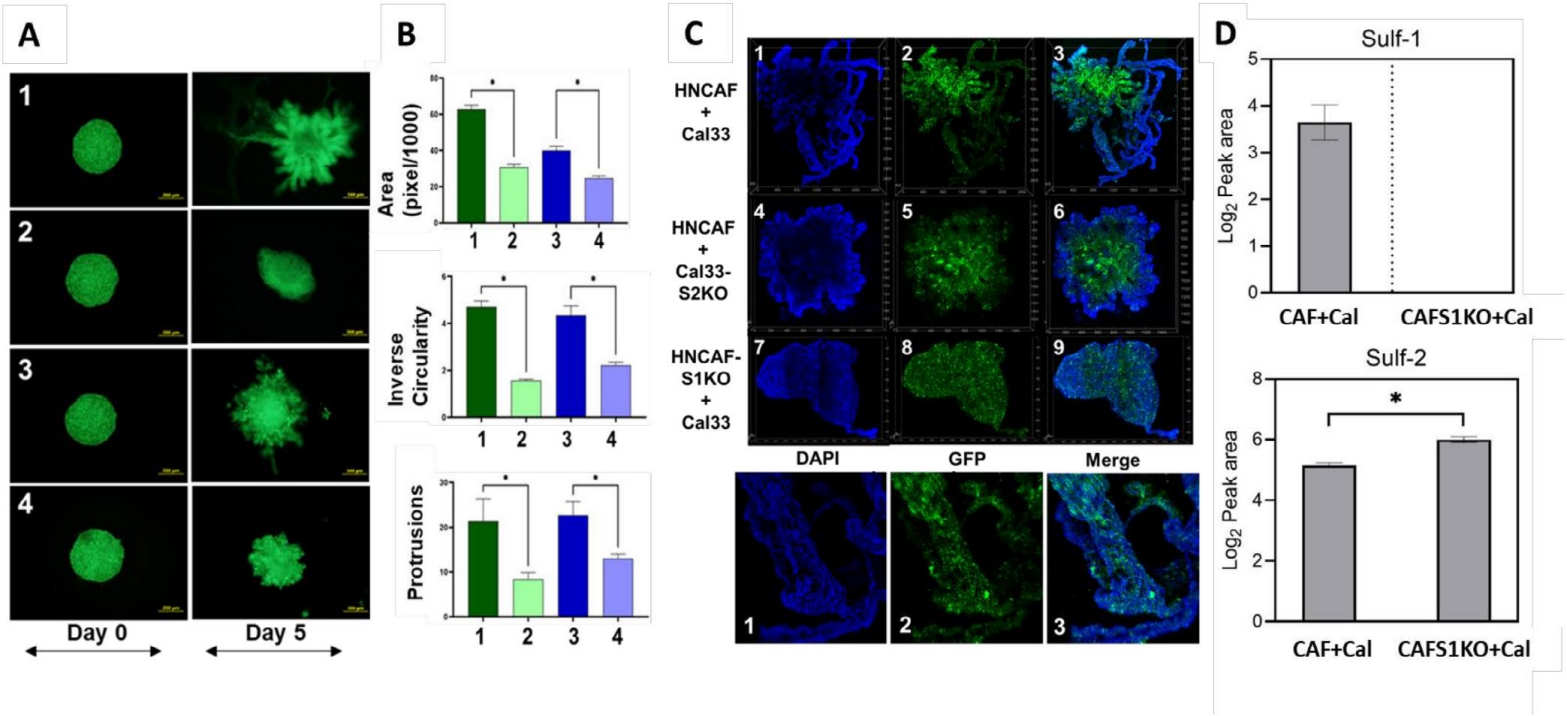
Impact of SULF1KO on spheroid invasion. (A) The left column shows images of coculture spheroids (3000 cells of each type) in Matrigel on the day of embedding (Day 0); the right column shows the spheroids on Day 5: (1) Cal33 + HNCAF37 (2) Cal33 + S1KOCAF (3) SCC35 + HNCAF37 (4) SCC35 + S1KOCAF. Images, taken under 5X magnification, show green fluorescence (FL) of the labeled Cal33 and SCC35 cells; CAF are not labeled. (B) The graphs represent the median ± SD (*n* = 3) for the area (top), inverse circularity (middle) and protrusions (bottom) of the coculture spheroids on day 5. Statistical significance was assessed using one‐way ANOVA. * indicates *p* ≤ 0.05. (C) Structure and spatial distribution of cells in a 3D coculture model of Cal33 (GFP) and HNCAF37 cells captured by two‐photon confocal laser scanning microscopy. Cancer cells are labeled with GFP (green), cell nuclei are stained with DAPI (blue), and the columns show individual colors and their overlay. The three images at the bottom represent magnification of the top row (Cal33 + HNCAF37) to highlight the presence of green cancer cell projections on the blue tracks. (D) Relative abundance of Sulf‐1 and Sulf‐2 proteins in HNCAF+Cal33 cocultured spheroids compared to HNCAFS1KO + Cal33.

Our recent studies of OSCC tumors documented the enrichment of SULF1^+^ CAFs in tumors of patients with poor prognosis [12, 15]. We have noticed high SULF1 expression in multiple types of tumors and hypothesized that the SULF1^+^ CAFs serve as its supply. Recent papers confirm that SULF1+ CAFs affect outcomes of lung [19] or colorectal cancers [20]. SULF1 is invariably present in the gene‐sets characterizing various types of myofibroblastic CAF typically associated with poor survival [7, 16, 21]. The CAF affect multiple critical cell communications, including angiogenesis, node invasion, or immune suppression [5, 7, 20]. The activity of the heparan 6‐O‐endosulfatases is expected to affect all these processes by adjusting the distribution and activity of multiple HS‐binding growth factors, morphogens, cytokines, or chemokines [22, 23]. The molecular mechanisms underlying these phenomena warrant further investigation in appropriate models incorporating the immune and other relevant cell types. Here we demonstrate that SULF1‐KO in primary HNSCC CAF significantly impairs cellular motility and invasive potential of cancer cells. Migration of the cancer cells is stimulated by the conditioned media of the HNCAF37 in a SULF1‐dependent manner (Figure 1), and invasion of the cancer cells into Matrigel in a spheroid coculture model is abrogated by the SULF1 KO in the HNCAF37 cells (Figure 2). Notably, the inverse circularity measurements and structural evaluations using confocal microscopy support the notion that CAF‐derived Sulf‐1 modulates extracellular matrix remodeling, thereby creating a permissive niche for tumor progression. The decreased invasion observed in Sulf1‐KO conditions suggests that targeted inhibition of Sulf‐1 may serve as a viable therapeutic strategy to attenuate stromal‐driven metastasis. Given the significant role of CAFs in modulating the tumor microenvironment, emerging therapeutic strategies targeting CAFs have garnered substantial interest. It is interesting to note that the SULF2 KO in the Cal33 cancer cells also limits tumor cell growth and limits cell invasion. The SULF1 and SULF2 isoforms are both secreted and have the same in vitro activity using various artificial substrates. We do not know how their activities differ in vivo but it has been shown that they strongly (if non‐covalently) associate with cell membranes [22, 24]. The fact that they are produced by different cell types suggests that the SULF1 will regulate the local microenvironment of the CAF more than the SULF2 which will be active closer to the tumor cells. However, both enzymes promote tumor growth and invasion and are associated with poor survival outcomes [12, 25]. These results suggest that targeting the SULF enzymes may be a viable approach for limiting tumor progression and metastasis in HNSCC and other malignancies, especially in the context of (neo)adjuvant therapy. Our observations suggest that incorporating Sulf‐1 analysis into diagnostic frameworks could enhance precision medicine approaches in HNSCC.

While our study provides robust insights into the role of SULF1 in CAF‐mediated invasion, certain limitations must be acknowledged. First, the impact of SULF1 inhibition on immune cell recruitment and angiogenesis was not explored in this model. To further clarify the mechanistic basis of SULF1‐driven stimulation of cancer cells, it would be informative to perform gain‐of‐function studies by knocking in SULF1 in HNCAFs, followed by analysis of growth factors and cytokine profiles in CAF‐conditioned media, as well as downstream signaling pathways in cancer cells exposed to SULF1‐expressing versus SULF1‐knockout CAFs. Additionally, further validation in vivo is warranted and requires careful study design. Expanding this study to include patient‐derived xenografts (PDXs) or organoid models may offer additional translational relevance. However, our study provides strong evidence that Sulf1^+^ CAFs facilitate stromal invasion and metastatic progression in HNSCC and justifies further exploration of the SULF enzymes as diagnostic and therapeutic targets. Future studies should focus on elucidating the downstream effectors of Sulf1 activity and identifying potential small‐molecule inhibitors capable of selectively modulating its function within the stroma.

## Author Contributions

Study design: R.G.; primary cell lines: L.A., cell model experiments: P.M., J.B., A.P.; data analysis: P.M., J.B., A.P.; writing – original draft: P.M.; review and editing: R.G., J.B., A.P., L.A. Corresponding author: R.G. All authors have read and agreed to the published version of the manuscript.

## Funding

This work was supported in part by a United States of America National Institutes of Health grant R01CA238455 to R. G. Further support was received from the NIH Office of the Director grant 1S10OD028623‐01A1 to R.G, S10OD032420 to Anastas Popratiloff, and Georgetown University Lombardi Comprehensive Cancer Center Support Grant from National Cancer Institute 2P30CA051008. The content is solely the responsibility of the authors and does not necessarily represent the official views of the National Institutes of Health.

## Ethics Statement

The study was conducted in accordance with the Declaration of Helsinki and approved by the Research Ethics Board of the Princess Margaret Cancer Centre, Toronto, CA (protocol 08‐0888‐T) for studies involving humans.

## Conflicts of Interest

The authors declare no conflicts of interest.

## Supporting information


**Figure S1:** Inference of Crispr Edits (ICE) analysis of SULF1 knockout HNCAF37 cells.
**Figure S2:** Linear‐scale visualization of viable cell growth over 4 days for HNCAF37 vs. CAFS1KO.

## Data Availability

All data underlying the results are available as part of the article and its supporting information files.
