# Supplementary material for: SULF1 in Cancer Associated Fibroblasts Promotes Invasion in Head and Neck Cancer Cell Lines
Source: Cancer Med. 2026 Jan 18;15(1):e71540. doi: 10.1002/cam4.71540 (PMC12812489; doi:10.1002/cam4.71540)
Supplement: Supplementary file 1 — Figure S1: Inference of Crispr Edits (ICE) analysis of SULF1 knockout HNCAF37 cells. Figure S2: Linear‐scale visualization of viable cell growth over 4 days for HNCAF37 vs. CAFS1KO. [file CAM4-15-e71540-s001.docx]

**SULF1 in cancer associated fibroblasts promotes invasion in head and neck cancer cell lines**

Pritha Mukherjee^1,2^, Julius Benicky^1,2^, Aswini Panigrahi ^1,2^, Laurie Ailles^3^, Radoslav Goldman^1,2,4^

1 Department of Oncology, Lombardi Comprehensive Cancer Center, Georgetown University,
Washington, DC 20057, USA

2 Clinical and Translational Glycoscience Research Center, Georgetown University,
Washington, DC 20057, USA

3 Department of Medical Biophysics, University of Toronto, Toronto, ON M5G 1L7, Canada

4 Department of Biochemistry and Molecular & Cellular Biology, Georgetown University,
Washington, DC 20057, USA

Corresponding author email: rg26@georgetown.edu

METHODS

Generation of Crispr/Cas9 SULF1 knockout cells.

SULF1-deficient primary human HNCAF37 cells were generated by Crispr/Cas9 gene editing using SULF1-specific Gene Knockout Kit v2 (Synthego, Redwood City, CA) as described previously (Reference 18). Briefly, ribonucleoprotein complexes consisting of 180 pmoles of SULF1-targeting synthetic sgRNAs and 20 pmoles of S. pyogenes Cas9 nuclease (Synthego) were nucleofected into 5x10^5^ HNCAF37 cells in Nucleofection Kit V Complete Solution (Lonza), using Nucleofector 2 (Lonza) and X-005 program according to the manufacturer’s instructions. The knockout efficiency was determined by Inference of CRISP Edits (ICE) analysis as described (Reference 18). Briefly, the targeted region of genomic DNA from Crispr/Cas9 edited and wild-type cells was amplified by PCR using Phusion Plus PCR Master Mix (Thermo Scientific), 100 ng of genomic DNA and 0.5 µM of each primer 5’ TCTGCCAGCTTATGTGCCAA 3’ (forward) and 5’ GTGCAAAGCCTCAGAATGTCT 3’ (reverse). The amplified DNA was submitted to Sanger sequencing (Genewiz, Chelmsford, MA) using sequencing primer 5’ CTTATGTGCCAATACTGACTTATTTGTAGC 3’. Trace files were uploaded to ICE Analysis Tool (Synthego, https://ice.synthego.com/#/) for analysis. The SULF1-targeted HNCAF37 cells had indel efficiency 99% (R^2^ 0.92, knockout score 90) (Figure S1). Knockout of the enzyme was verified by targeted LC-MS/MS-PRM assays for both SULF1 and SULF2 proteins

SULF1:

sgRNA_1: TGCTCTGGTTTTGGCTGTCC

sgRNA_2: GTCAGATCCCCGAGGTTCAG

sgRNA_3: GCTTACCGATGATCAAGATG

Relative Quantification of proteins

Cancer and CAF cells were co-cultured for 72 hours, and ~25,000 cells were washed three times with PBS. The cells were lysed in 100 µl of 0.1% DDM with sonication. The proteins were then reduced, alkylated, and digested with LysC-Trypsin in Barocycler. The resulting peptides were desalted with C_18_ ZipTip. The samples in triplicate were analyzed by diaPASEF LC-MS essentially as described by using TimsTOF Ultra mass spectrometer (Reference 15). Data analysis was performed using Spectronaut 19 software, and the peak areas of Sulf-1 and Sulf-2 proteins were extracted. Their relative abundance and statistical significance (t-test) was plotted using GraphPad Prism Software (v 10.5)

Spheroid co-culture

Spheroids were cultured as described previously (13, Mukherjee et al 2023). Briefly, HNSCC and HNCAF37 cells were equally mixed (3 × 10^4^ cells/mL for each cell type) and 100 µl of the mixture was dispensed into ultra-low attachment 96-well round bottom plate (Corning, Kennebunk, ME), allowed 24 hrs to form spheroid followed by Matrigel embedding. The embedded spheroids were cultured for 5 days and imaged using Olympus IX71 inverted microscope (Olympus, Tokyo, Japan). Digital images of spheroids were analyzed by FIJI image analysis software (1) using INSIDIA macro for spheroid invasion analysis (2).

Migration assays conditions

Wild-type HNCAF37 and SULF1-knockout CAFs were seeded at identical initial densities (depending on the flask dimensions) and allowed to grow to comparable confluency (80-85%) prior to conditioning. Conditioned media were then collected over the same incubation period (24h), clarified by centrifugation, and applied to cancer cells at equivalent volumes.

References

1. J. Schindelin, I. Arganda‐Carreras, E. Frise, et al., “Fiji: An Open‐Source Platform for Biological‐Image Analysis,” Nature Methods 9, no. 7 (2012): 676–682.
2. C. Moriconi, V. Palmieri, R. Di Santo, et al., “INSIDIA: A Fiji Macro Delivering High‐Throughput and High‐Content Spheroid Invasion Analysis,” Biotechnology Journal 12, no. 10 (2017): 1700140.


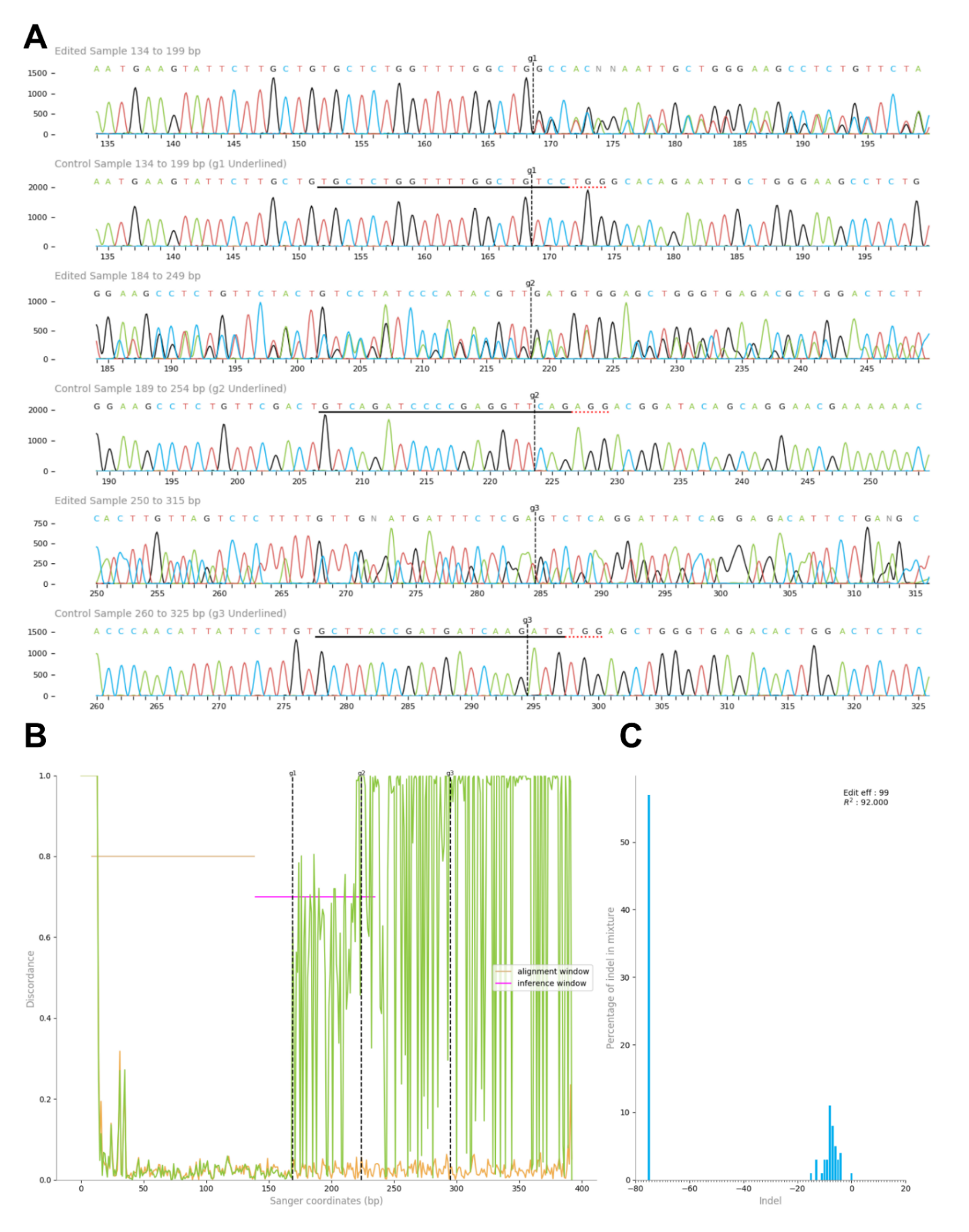


**Figure S1**. Inference of Crispr Edits (ICE) analysis of SULF1 knockout HNCAF37 cells. (A) Comparison of wild-type (bottom) and knockout (upper) trace files obtained by Sanger sequencing of the edited genomic DNA region. (B) Discordance plot showing the base-by-base level of disagreement between the non-edited wild-type (orange line) and the edited sample (green line) in the inference window around the edited region. (C) Histogram showing the percentual distribution of indel sizes in the edited population. Three sgRNAs are referred to as g1, g2, and g3.


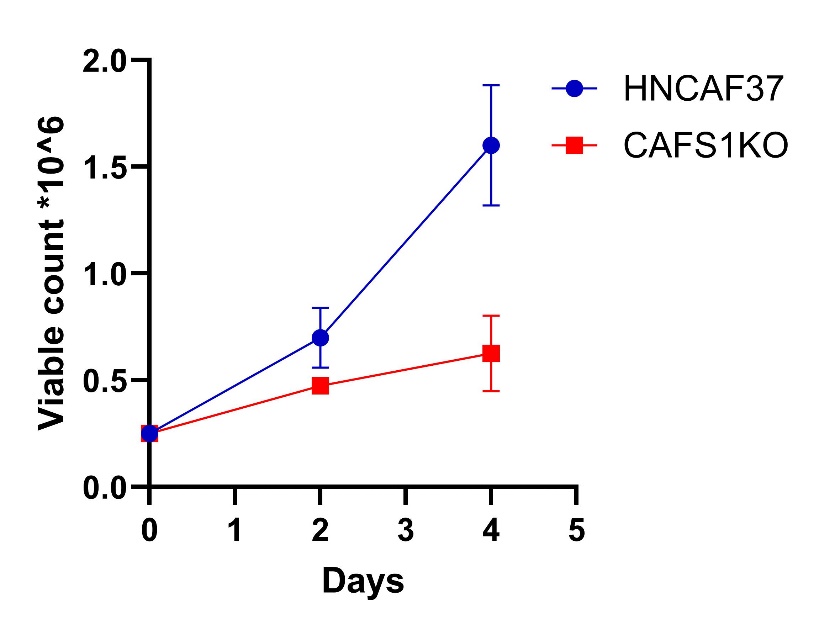


**Figure S2. Linear-scale** visualization of viable cell growth over 4 days for **HNCAF37** vs. **CAFS1KO.** Day 0**:** Both cell types start with ~0.25 × 10⁶ viable cells. HNCAF37**:** Shows a marked increase to ~1.7 × 10⁶ by Day 4. CAFS1KO**:** Shows modest growth, reaching ~0.6 × 10⁶ by Day 4. Viable count calculated using trypan blue and countess chamber slides. n=3 replicates for each sample.
